# Proteomic analysis of saliva reveals changes in proteomic profiles during colorectal cancer in an Iranian cohort

**DOI:** 10.3389/fonc.2026.1854834

**Published:** 2026-06-24

**Authors:** Sama Rezasoltani, Ali Biabani, Bente Siebels, Hamid Asadzadeh Aghdaei, Georg Conrads, Hartmut Schlüter, Mohammad Mehdi Feizabadi

**Affiliations:** 1 Section Mass Spectrometry and Proteomics, University Medical Center Hamburg-Eppendorf (UKE), Hamburg, Germany; 2 Division of Oral Microbiology and Immunology, Department of Operative Dentistry, Periodontology and Preventive Dentistry, Rheinisch-Westfälische Technische Hochschule (RWTH) University Hospital, Aachen, Germany; 3 Basic and Molecular Epidemiology of Gastrointestinal Disorders Research Center, Research Institute for Gastroenterology and Liver Diseases, Shahid Beheshti University of Medical Sciences, Tehran, Iran; 4 Gastroenterology Department, Lismore Base Hospital, North New South Wales Local Health District, Lismore, NSW, Australia; 5 Department of Microbiology, School of Medicine, Tehran University of Medical Sciences, Tehran, Iran; 6 Thoracic Research Center, Imam Khomeini Hospital Complex, Tehran University of Medical Sciences, Tehran, Iran

**Keywords:** biomarkers, colorectal cancer, early detection, LC–MS/MS, proteomics, salivary proteins

## Abstract

**Introduction:**

Colorectal cancer (CRC) is common and associated with poor survival, and early detection is pivotal to improving patient outcomes. Therefore, identifying reliable biomarkers reflecting early-stage tumor development is of major clinical importance. This study aimed to identify salivary proteins with potential as non-invasive biomarkers associated with early-stage CRC.

**Methods:**

A total of 104 saliva samples were collected from individuals undergoing CRC screening, including patients diagnosed with CRC stage I and non-cancer controls (NCC), at Taleghani Hospital, Tehran, Iran, in this case–control study. A quantitative label-free proteomics approach was applied to determine global proteome differences between CRC patients and NCCs and to identify proteins with altered abundance. Proteins were extracted from saliva samples, digested with trypsin, and peptides were analyzed using LC–MS/MS on an Orbitrap Fusion mass spectrometer.

**Results:**

A total of 2,456 salivary proteins were identified, of which 181 showed significantly different abundance between CRC patients and NCCs. Unsupervised clustering demonstrated partial separation of groups with influence from demographic variables. Members of the small proline-rich protein (SPRR) family emerged as consistent core markers distinguishing CRC from NCCs, while additional immune- and metabolism-associated proteins contributed to group differentiation. Demographic subgroup analyses, including age-, sex-, and smoking-stratified comparisons, revealed subgroup-associated differences in protein abundance patterns; however, several CRC-associated alterations remained detectable across subgroups, including middle-aged individuals. Gene set enrichment analysis indicated suppression of cytoskeletal and epithelial structural pathways alongside enrichment of protease regulatory pathways. Ingenuity Pathway Analysis identified an interconnected immune–metabolic network characterized by inflammatory signaling and oxidative stress–related processes. These alterations indicate immune-related and metabolic signaling changes associated with CRC.

**Discussion:**

This study supports salivary proteomics as a non-invasive approach for early CRC detection, and the observed proteomic signatures may reflect systemic tumor–immune interactions associated with CRC. Data are available via ProteomeXchange with identifier PXD078610.

## Background

Colorectal cancer (CRC) persists as a significant healthcare challenge, comprising approximately 6.1% of all cancers globally (1). The International Agency for Research on Cancer provides estimates of CRC incidence and mortality across world regions. In 2020 alone, over 1.9 million new cases of CRC and more than 930,000 CRC-related deaths were estimated worldwide (2). When CRC is detected in its early stages (localized, stages 0 to II), the five-year survival rate exceeds 80%. However, this rate drops dramatically in advanced stages, particularly stage IV disease (3). Compounding the issue, many instances of CRC stem from initially benign colonic adenomas (4). Given that the survival rate steadily declines with advancing cancer stages at diagnosis, the imperative of early detection and removal of these precancerous adenomas cannot be overstated (5). Consequently, widespread population screening and prevention initiatives are strongly advocated globally (6). Contemporary population-based CRC screening programs predominantly rely on fecal immunochemical testing (FIT) to identify high-risk individuals, a method credited with lowering CRC mortality rates. However, FIT’s sensitivity is limited, yielding inconsistent outcomes across diverse populations (7). Furthermore, this approach often leads to unnecessary colonoscopies, an invasive and costly procedure (8). Consequently, there is a pressing demand for more precise and non-invasive screening methodologies to identify individuals in the early stages of CRC.

Today, the potential value of saliva in the early diagnosis of oral diseases, as well as cancers, diabetes, and other systemic disorders is increasingly recognized (9–11). With the rapid advancement in salivaomics, saliva is a rich source of biomarkers (11). Proteomic analyses have identified over 3,700 human proteins in saliva and more than 2,000 detectable microbial proteins; however, the total proteomic diversity likely exceeds these numbers due to methodological and detection constraints (12). Recent studies have uncovered shifts in both microbial patterns and protein profiles in saliva, particularly in relation to various diseases, notably cancers (13, 14). Meanwhile, CRC is strongly influenced by epigenetic alterations, with accumulating evidence highlighting the role of gut microbiota in disease development (15, 16).

Our recent studies have revealed distinct microbial patterns in the saliva of individuals with CRC compared to non-cancer controls (NCCs) within the Iranian population (17, 18). Our findings suggest that a range of microbial biomarkers may serve as candidates for the early detection of CRC. Building upon our previous investigations, this study aims to characterize the salivary protein profiles of CRC patients compared to NCCs in an Iranian cohort. Our objective is to determine whether the protein patterns in the saliva of CRC patients differ significantly from those of NCCs and to explore the feasibility of utilizing salivary protein biomarkers as candidates for CRC screening. We also examine demographic characteristics of patients and NCCs. This emphasis is crucial, as factors such as sex, age, lifestyle, diet, overall health, and geographic context might influence salivary protein profiles.

## Methods

### Study population

The present study was a case-control analysis, with all saliva samples procured from patients referred for standard screening colonoscopy between February 2020 and January 2022 at Taleghani Hospital in Tehran, Iran. Enrolled participants, aged ≥18 years, were recruited from patients referred to Taleghani Hospital from different regions of Iran. A total of 104 saliva samples were collected, comprising 51 NCCs and 53 CRC cases stage I, prior to colonoscopy procedures. Participant status (CRC vs. NCCs) was determined by colonoscopy and subsequently confirmed by histopathological examination. Patients exhibiting symptoms such as rectal bleeding, changes in bowel habits, anemia, and abdominal pain were included in the study, while exclusion criteria comprised individuals: 1) who had taken antibiotics within the past three months (with the exception of one participant who had used antibiotics for less than one week), 2) who had regularly used probiotics within the past three months (with the exception of one NCC who had used probiotics for one week), 3) who had undergone invasive medical interventions within the same period; 4) with a previous history of CRC, inflammatory or infectious intestinal diseases; 5) with gastrointestinal disorders like inflammatory bowel disease, irritable bowel syndrome, liver disorders, or non-alcoholic fatty liver disease; and 6) categorized as high-risk for CRC, such as those with familial adenomatous polyposis or other hereditary cancer syndromes. Demographic and clinical data, including age, sex, profession, physical activity, disease or surgical history, family history, smoking status, alcohol consumption, dietary patterns, viral infection status, probiotic use, and antibiotic usage, were collected through standardized questionnaires. NCC participants were chosen based on normal colonoscopy results and a negative personal and family history of gastrointestinal diseases. Patients were approached during their initial hospital visit to explain the research protocol and obtain informed consent. Approval for this case-control study was granted by the Clinical Research Ethics Committee of Shahid Beheshti University of Medical Sciences and the Ethics Committee of Taleghani Hospital, Tehran, Iran (IR.SBMU.RIGLD.REC.1398.039).

### Human saliva sampling

Saliva specimens were prospectively collected between 08:00 - 12:00, with participants instructed to abstain from eating or drinking for at least 2 h prior to sampling. Each saliva sample was collected into a 20 mL Falcon tube kept on ice. Approximately 1–2 mL of unstimulated saliva was collected over a period of 5–10 min. Following collection, the specimens were promptly transferred to new 2 mL microcentrifuge tubes and immediately frozen at −80 °C to preserve their integrity for subsequent evaluation (19).

### Protein extraction and tryptic digestion

500 µL of saliva were mixed with an equal volume of 6 M urea buffer, and 2 µL of Sera-Mag™ carboxylate-modified hydrophobic and hydrophilic magnetic beads were added. Proteins were reduced using 0.5 µL of 1 M dithiothreitol (DTT) for 30 min at 60 °C, followed by alkylation with 2 µL of 0.5 M iodoacetamide for 30 min at 37 °C in the dark. Proteolytic digestion was initiated by adding 1 µL of a trypsin solution (0.5 µg/µL) to each sample, prepared by dissolving 20 µg of trypsin in 40 µL of 50 mM ammonium bicarbonate. Digestion proceeded overnight at 37 °C using a shaker. Following enzymatic digestion, the samples were washed two times with 100% acetonitrile (ACN). Based on the SP3 protocol by Hughes et al. (2019), following proteolytic digestion the magnetic beads with bound peptides were washed twice, with 70% ethanol to remove residual contaminants, ensuring that peptides remained immobilized on the beads during the cleanup step (20). Peptides were eluted with 50 µL of a solution containing 2% dimethyl sulfoxide (DMSO) and 1% formic acid (FA). The eluates were dried using vacuum centrifugation, reconstituted in 0.1% FA, and stored at -20 °C until LC-MS/MS analysis (20–22).

### LC-MS/MS analysis using data-dependent acquisition

Chromatographic separation of peptides was achieved with a two-buffer system (buffer A: 0.1% FA in H_2_O, buffer B: 0.1% FA in ACN) on a nano-UHPLC (Dionex Ultimate 3000 UHPLC system, Thermo Fisher). Attached to the UHPLC was a peptide trap (100 µm x 20 mm, 100 Å pore size, 5 µm particle size, C18, nanoViper, Thermo Fisher) for online desalting and purification, followed by a 25 cm C18 reversed-phase column (75 µm x 250 mm, 130 Å pore size, 1.7 µm particle size, peptide BEH C18, nanoEase, Waters). Peptides were separated using an 80-minute method with a linear increase in ACN concentration from 2% to 30% ACN over 60 minutes, followed by high organic wash and re-equilibration. MS/MS measurements were performed on an Orbitrap Fusion Tribrid mass spectrometer (Thermo Fisher). Eluting peptides were ionized using a nano-electrospray ionization (nano-ESI) source with a spray voltage of 1.8kV and analyzed in data-dependent acquisition (DDA) mode. For each MS1 scan, ions were accumulated for a maximum of 120 ms or until an AGC target of 2 × 10^5^ ions was reached. Fourier-transform-based mass analysis in the Orbitrap covered m/z 400–1,300 at a resolution of 120,000 at m/z = 200. Peptides with charge states between 2+ to 5+ above an intensity threshold of 1,000 were isolated within a 1.6 m/z isolation window in Top Speed mode for 3 s per cycle and fragmented with a normalized collision energy of 30% using higher-energy collisional dissociation (HCD). MS2 scans were acquired in the ion trap at a rapid scan rate, covering a mass range starting at m/z 120 and accumulated for 60 ms or to an AGC target of 1 × 10^5^. Dynamic exclusion was set to 30 s (23, 24).

### Data analysis

Raw MS data were processed using Proteome Discoverer (PD) version 3.1.0.638 (Thermo Fisher Scientific), with MS/MS spectra searched against the *Homo sapiens* reference proteome (UniProtKB/Swiss-Prot canonical, TaxID 9606, release 2023-11-08). A static modification of cysteine (carbamidomethylation) was specified, and variable modifications included methionine oxidation and pyroglutamate formation. Trypsin was specified as the proteolytic enzyme, allowing a maximum of two missed cleavages. A strict false discovery rate (FDR) threshold of 1% was applied at the peptide-spectrum match (PSM) and protein levels to ensure high-confidence identifications. Label-free quantification (LFQ) was performed using the Minora Feature detector. The resulting protein abundances were log2-transformed and normalized using column-wise median normalization in Perseus (version 2.0.11) (23, 24). The large number of samples made it necessary to split the samples into different preparation and LC-MS/MS cohorts. Resulting batch effects were removed with the Batch-Effect Reduction Trees (BERT) algorithm, using the reference based method, which operates on data matrices with missing values and thus avoids listwise deletion (25). For statistical analysis, a two-sided Student’s t-test was applied. P-values were adjusted for multiple testing using the Benjamini-Hochberg false discovery rate (FDR) procedure. Proteins with an adjusted p-value (q-value) < 0.05 were considered statistically significant. Visualizations were performed in R Studio (Version 2025.05.0 + 496) using ggplot and pHeatmap. Gene set enrichment analysis (GSEA) was performed to identify biological pathways and functional categories associated with differential protein abundance between groups and was conducted in R using the clusterProfiler package (26). Gene sets were obtained from the Gene Ontology (GO) database, including the Biological Process (BP), Cellular Component (CC), and Molecular Function (MF) ontologies. Gene sets containing fewer than 15 or more than 500 genes were excluded from the analysis. Statistical significance was assessed using Benjamini-Hochberg multiple testing correction, and enriched terms were evaluated based on the false discovery rate (FDR) q-value. Ingenuity pathway analysis (IPA, version 153384343, QIAGEN) was performed using proteins with nominal p-values < 0.05 and an absolute log2 fold change ≥ 1.0.

## Results

### Comparative analysis of demographic characteristics in CRC patients

**Table 1** summarizes the demographic and clinical characteristics of the study participants. As expected, CRC patients were significantly older than NCCs (P < 0.001). Significant differences were also observed in employment status, family history of CRC, disease or surgical history, and physical activity (all P < 0.05). In contrast, no statistically significant differences were detected between groups with respect to sex, viral infection status, smoking, alcohol consumption, dietary pattern, or regular probiotic and antibiotic use. Overall, the two groups were comparable in major lifestyle-related variables, while age and selected clinical characteristics differed between CRC patients and NCCs.

**Table 1:** Baseline demographic and clinical characteristics of colorectal cancer (CRC) patients and non-cancer controls (NCCs).

Variable	Category	CRC stage I (n=53)	NCC (n=51)	P-value
Age		59.19 ± 13.95	36.20 ± 11.99	<0.001
Sex	Female	29 (54.72)	31 (60.78)	0.557
	Male	24 (45.28)	20 (39.22)	
Profession	No	43 (81.13)	12 (23.53)	<0.001
	Yes	10 (18.87)	39 (76.47)	
Family history	No	28 (52.83)	40 (78.43)	0.008
	Yes	25 (47.17)	11 (21.57)	
Disease or surgical history	No	26 (49.06)	36 (70.59)	0.029
	Yes	27 (50.94)	15 (29.41)	
Viral infection	No	52 (98.11)	51 (100.00)	1.000
	Yes	1 (1.89)	0 (0.00)	
Smoking	No	43 (81.13)	46 (90.20)	0.266
	Yes	10 (18.87)	5 (9.80)	
Alcohol consumption	No	50 (94.34)	46 (90.20)	0.484
	Yes	3 (5.66)	5 (9.80)	
Physical activity	No	46 (86.79)	20 (39.22)	<0.001
	Yes	7 (13.21)	31 (60.78)	
Dietary pattern	High consumption of meat	14 (26.42)	5 (9.80)	0.081
	High consumption of vegetables and fruits	5 (9.43)	8 (15.69)	
	All food (vegetable and meat)	34 (64.15)	38 (74.51)	
Regular probiotic	No	53 (100.00)	50 (98.04)	0.490
	Yes	0 (0.00)	1 (1.96)	
Antibiotic usage	No	52 (98.11)	51 (100.00)	1.000
	Yes	1 (1.89)	0 (0.00)	

Age is expressed as mean ± SD; categorical variables are presented as n (%).

### Differential proteomic analysis to reveal biomarker candidates

**Figure 1:**
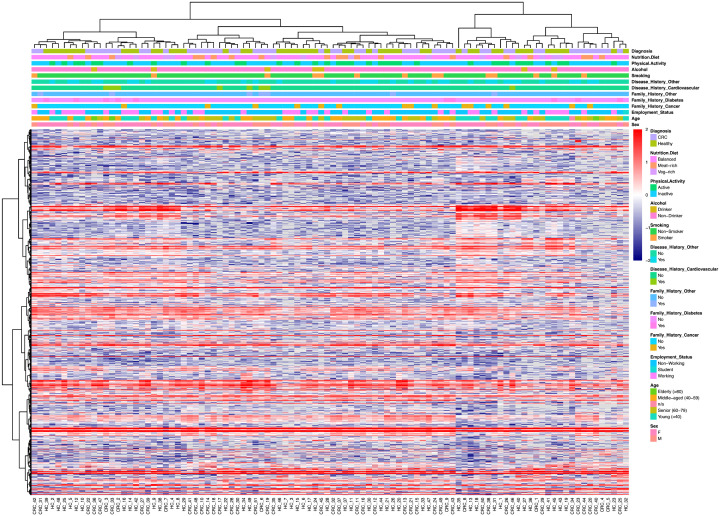
Clustered heatmap of differentially abundant proteins in saliva samples from colorectal cancer (CRC) patients and non-cancer controls (NCC). Hierarchical clustering was performed on both proteins (rows) and samples (columns) using Pearson-correlation distance with average linkage. Rows represent proteins; columns represent individual samples. Values are mean-centered per protein (row-wise; each protein’s across-sample mean was subtracted); red indicates higher abundance and blue indicates lower abundance. The top annotation bars denote sample annotations including demographic and clinical variables (e.g., age, sex, smoking). A partial, but not complete, separation between CRC and NCC was observed, consistent with considerable inter-individual heterogeneity in salivary proteomes. In this figure, the label “Healthy” refers to non-cancer controls (NCCs).

In total, 2,456 proteins were identified and quantified across saliva samples from CRC patients and NCCs. Differential abundance analysis revealed 181 proteins that were significantly altered between the two groups. To evaluate whether these proteins could distinguish CRC patients from NCCs, their expression profiles were visualized across all samples. The resulting heatmap (**Figure 1**) demonstrated partial separation between CRC and NCC samples, although considerable heterogeneity was observed within both groups.

**Figure 2:**
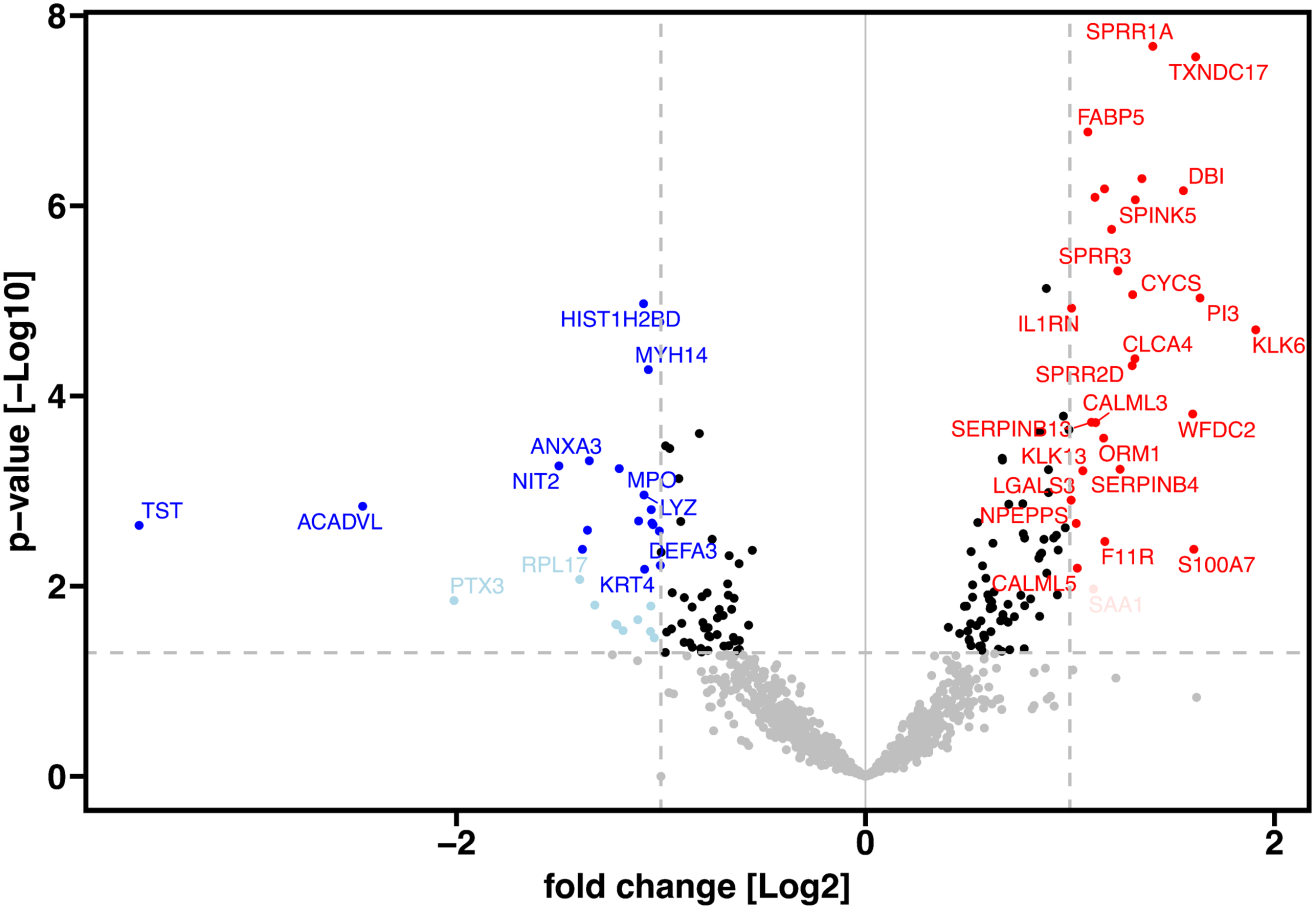
Volcano plot shows differentially abundant salivary proteins between colorectal cancer (CRC) patients and non-cancer controls (NCCs). The x-axis represents the log2 fold change (CRC vs. NCCs), where positive values indicate higher abundance in CRC samples, and the y-axis represents −log10-transformed nominal p-values. Differential abundance analysis was adjusted for age, sex, and smoking status, and Benjamini–Hochberg false discovery rate (FDR)-adjusted q-values were used to correct for multiple testing. Red dots indicate proteins with q < 0.05 and |log2FC| ≥ 1 (≥2-fold) showing higher abundance in CRC, whereas blue dots indicate proteins with q < 0.05 and |log2FC| ≥ 1 showing lower abundance in CRC. Pink and pale blue dots represent proteins with p < 0.05 and |log2FC| ≥ 1 that did not remain significant after FDR correction. Black dots indicate proteins with p < 0.05 but |log2FC| < 1, while gray dots represent non-significant proteins (p ≥ 0.05). Vertical dashed lines indicate |log2FC| = 1, and the horizontal dashed line indicates p = 0.05 (−log10(0.05) ≈ 1.3).

Using a volcano plot, we visualized the log_2_ fold-change (log_2_FC) in protein abundance between CRC and NCC against the corresponding -log_10_ p-values (**Figure 2**). Proteins with an absolute log2 fold change ≥ 1 (≥ 2-fold change) and an q-value < 0.05 were considered differentially abundant (27). Several proteins exhibited markedly higher or lower abundance in CRC patients compared with NCCs. Notably, multiple members of the small proline-rich protein (SPRR) family, including SPRR1A, SPRR3, and SPRR2D showed significantly higher abundance in CRC. Increased abundance was also observed for thioredoxin domain-containing protein 17 (TXNDC17), diazepam binding inhibitor (DBI), peptidase inhibitor 3 (PI3/Elafin), kallikrein-related peptidase 6 (KLK6), WAP four-disulfide core domain protein 2 (WFDC2), and fatty acid binding protein 5 (FABP5). For clarity, only selected representative proteins are labeled in the plot. Potential demographic influences, including age, sex, and smoking status, were considered during the differential-abundance analysis and further explored through subgroup analyses.

Prior to expansion of the study cohort, an initial exploratory analysis was performed using a smaller subset of samples (20 CRC and 20 NCCs) to assess the reproducibility of the proteomic workflow and the detectability of CRC-associated salivary protein alterations. Notably, several candidate proteins identified in the final cohort, including SPRR3, FABP5, DBI, PI3, WFDC2, and SOD1, were also consistently detected in this preliminary analysis. These exploratory findings are summarized in **Supplementary Table 1**.

**Figure 3:**
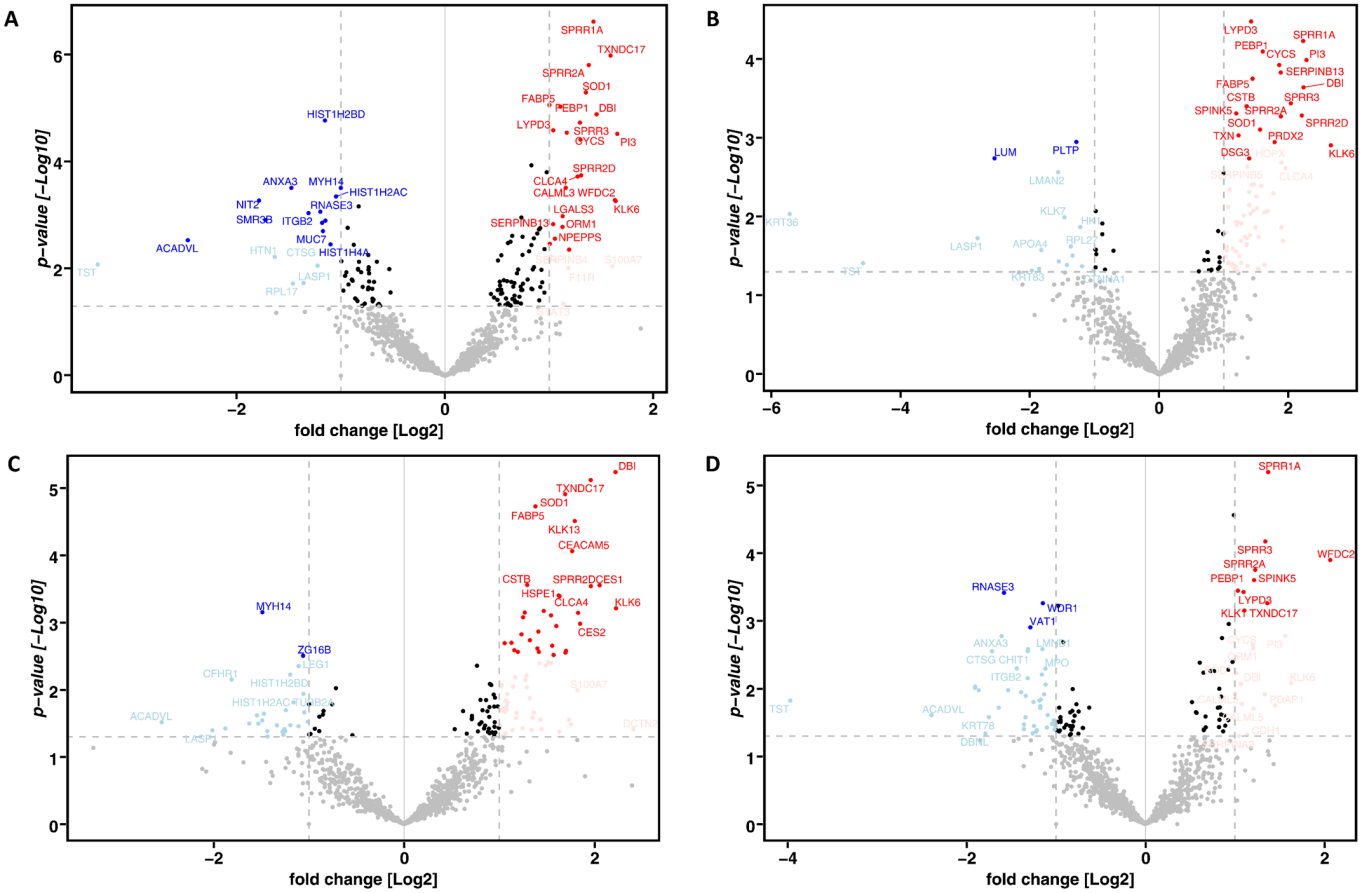
Volcano plots of differentially abundant salivary proteins between colorectal cancer (CRC) patients and non-cancer controls (NCCs), stratified by demographic variables. **(A)** Non-smokers, **(B)** middle-aged individuals (40–59 years), **(C)** males, and **(D)** females. Differential abundance analysis was performed using Student’s t-test followed by Benjamini–Hochberg false discovery rate (FDR) correction. Vertical dashed lines indicate |log2FC| = 1, and the horizontal dashed line indicates p = 0.05. Gray dots represent non-significant proteins; black dots indicate proteins with p < 0.05 but |log2FC| < 1; pale blue and pink dots represent proteins with >2-fold lower or higher abundance, respectively, based on unadjusted p-values; blue and red dots indicate proteins with >2-fold lower or higher abundance, respectively, that remained significant after FDR correction (q < 0.05).

To evaluate the potential influence of demographic variables on salivary protein patterns, subgroup analyses stratified by smoking status, age, and sex were performed (**Figure 3**). In the middle-aged subgroup (40–59 years; **Figure 3B**), several CRC-associated salivary protein alterations remained detectable, supporting that the observed proteomic differences were not solely attributable to age-related changes. Similar CRC-associated alterations were also observed across the smoking- and sex-stratified subgroups. In the non-smoker subgroup (**Figure 3A**), multiple proteins remained differentially abundant between CRC patients and NCCs. In the male subgroup (**Figure 3C**), proteins including CEACAM5, CSTB, SOD1, FABP5, DBI, and KLK13 demonstrated higher abundance in CRC samples. In the female subgroup (**Figure 3D**), several proteins exhibited higher abundance in CRC compared with NCCs, including members of the small proline-rich protein (SPRR) family (SPRR1A, SPRR3, and SPRR2A), as well as WFDC2 and TXNDC17. Overall, these findings suggest that the core CRC-associated salivary protein signature is detectable across demographic subgroups, including the middle-aged subgroup despite the overall age disparity between CRC patients and NCCs, while certain proteins may exhibit subgroup-specific abundance differences.

In this study, we identified candidates in the saliva of CRC patients that demonstrated significant alterations compared to NCCs. These proteins were analyzed and compared with those reported in previous CRC studies (**Table 2**). Our findings suggest that several of these differentially abundant proteins have been previously described in CRC, primarily in tissue or serum samples. Their detection in saliva in our cohort further supports their potential relevance as non-invasive biomarkers.

**Table 2:** Candidate salivary protein biomarkers identified in colorectal cancer (CRC) versus non-cancer controls (NCC), compared with biomarkers reported in previous CRC studies.

Protein name	Gene name	UniProt accession number	Mechanism in CRC	Sample source	Stage/phase of CRC	References
Small Proline-Rich Proteins (SPRR1A, SPRR3)	SPRR1A, SPRR3	P35321, P26447	Enhances cell migration, invasion, and metastasis in CRC.	Tissue	Metastatic CRC	(27, 63)
Diazepam Binding Inhibitor (DBI)	DBI	P07108	Involved in lipid metabolism; implicated in tumor progression.	Tissue	CRC progression	(64)
Kallikrein-Related Peptidase 6 (KLK6)	KLK6	Q92876	Associated with protease activity, promoting metastasis and poor prognosis in CRC.	Serum, tissue	Metastatic CRC	(45, 65)
S100A8/A9 (Calprotectin)	S100A8, S100A9	P05109, P06702	Linked to neutrophil deregulation, CRC progression, and inflammation.	Serum, tissue	CRC progression	(66, 67)
Superoxide Dismutase 1 (SOD1)	SOD1	P00441	Implicated in oxidative stress response and tumor suppression in CRC.	Serum	Early and late-stage CRC	(68, 69)
Peptidase Inhibitor 3 (Elafin, PI3)	PI3	P19957	Plays a role in inflammation and immune response, associated with metastasis in CRC.	Serum	Metastatic CRC	(70)
Aspartate Beta-Hydroxylase (ASPH)	ASPH	Q12797	Promotes cell migration and invasion, associated with metastatic CRC.	Tissue	Metastatic CRC	(71)

Notably, some of the protein biomarker candidates detected in CRC were also reported in other oro-gastrointestinal cancers, including oral squamous cell carcinoma (OSCC), esophageal cancer, gastric cancer, liver cancer, and pancreatic cancer. These shared biomarkers, summarized in **Table 3**, indicate potential overlap in molecular processes across GI malignancies. While these proteins may have broader relevance in cancer biology, our findings emphasize the importance of further evaluating CRC-associated markers to improve diagnostic specificity.

**Table 3:** Comparison of salivary protein biomarker candidates identified in this study for colorectal cancer (CRC) versus non-cancer controls (NCC), with reports in other gastrointestinal (GI) cancers.

Protein name	Gene name	UniProt accession number	Mechanism in cancer	Sample source	Disease	References
Alpha-1 Antitrypsin (A1AT)	SERPINA1	P01009, P02763	Protease inhibitor, tissue protection, modulating inflammation and immune responses.	Serum	Oral squamous cell carcinoma (OSCC)	(72)
Interleukin-1 Receptor Antagonist Protein (IL1RN)	IL1RN	P18510	Inhibits IL-1 signaling, suppressing inflammation and early carcinogenic events.	Tissue, Serum	OSCC	(73, 74)
Cystatin-B (CSTB)	CSTB	P04080	Inhibitor of cysteine proteases, increased cell proliferation and migration, reducing apoptosis.	Gastric cancer SGC-7901 cell line	Gastric cancer (GC)	(75)
Triosephosphate Isomerase (TPI)	TPI1	P60174	Involved in glycolysis; facilitating metabolic reprogramming in cancer cells, contributing to rapid cell proliferation.	Gastric cancer MGC-803 cell line	GC	(76)
Aflatoxin B1 Aldehyde Reductase (AKR7A2)	AKR7A2	Q43488	Detoxification of carcinogenic aldehydes, involved in resistance to chemotherapy, protection against oxidative stress.	Pancreatic ductal adenocarcinoma tissue, normal adjacent pancreatic tissue, pancreatic benign cystadenoma tissue	Pancreatic ductal adenocarcinoma	(77)
Annexin A7 (ANXA7)	ANXA7	P20073	Regulating cell proliferation, apoptosis, migration, and invasion facilitating tumor metastasis.	Cancer tissues and adjacent normal tissues	Various cancers including hepatocellular carcinoma	(78)
Sialic Acid Binding Ig-like Lectin 5 (SIGLEC5)	SIGLEC5	Q9Y336	Immune evasion, modulation of tumor microenvironment, regulation of immune response.	Tumor tissues, cell lines, patient samples	Various cancers including colorectal cancer (CRC)	(79)
Y Box Binding Protein 1 (YBX1)	YBX1	P67809	Involved in the regulation of oncogenes and tumor suppressors, promotion of metastasis, and resistance to therapy.	Tissue and cell lines	Various human cancers including pancreatic cancer and CRC	(80)

### Functional analysis of salivary protein alterations in CRC

**Figure 4:**
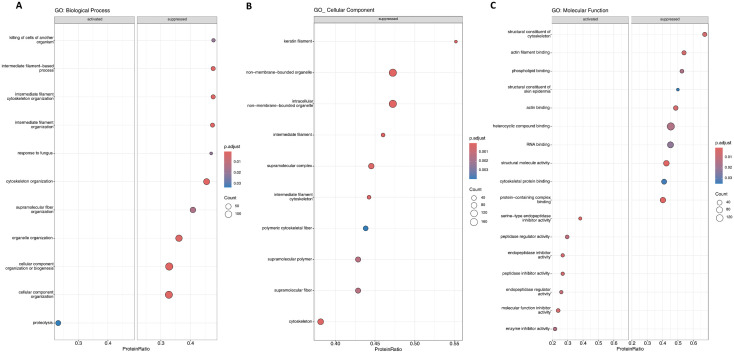
Gene Set Enrichment Analysis (GSEA) of differentially abundant salivary proteins between CRC patients and non-cancer controls (NCCs), categorized by Gene Ontology (GO) domains: **(A)** Biological Process (BP), **(B)** Cellular Component (CC), **(C)** Molecular Function (MF). Dot plots depict the top enriched terms, separated into activated (higher representation in CRC) and suppressed (lower representation in CRC) categories. Bubble size represents the number of proteins in each term (set size); the color gradient indicates adjusted p-values (q-values). Enrichment was performed using the GO database and visualized with dot plots.

To further elucidate the functional implications of salivary protein alterations between CRC patients and NCCs, gene set enrichment analysis (GSEA) was conducted across three major Gene Ontology (GO) categories, Biological Process (BP), Cellular Component (CC), and Molecular Function (MF), as shown in **Figures 4A–C**. Distinct patterns of enrichment were observed, with a predominant involvement of cytoskeleton-related pathways and proteolytic regulation (28, 29).

Within the BP category (**Figure 4a**), several terms were relatively decreased in CRC compared to NCCs, particularly those related to cytoskeletal and intermediate filament organization. These included intermediate filament organization, intermediate filament cytoskeleton organization, cytoskeleton organization, and supramolecular fiber organization, suggesting altered representation of structural protein programs in CRC saliva (28–30). Epithelial-associated processes, such as keratin-related pathways, were observed within this broader pattern of reduced structural organization (29, 30). Conversely, fewer but notable activated processes were detected, with proteolysis emerging as a consistently enriched biological process (**Figure 4A**), suggesting altered protease activity in CRC saliva (31).

Analysis of the CC category revealed that the most significantly affected compartments were structural elements associated with the cytoskeleton and filament-based assemblies (**Figure 4B**). Several cytoskeleton-related components, including intermediate filament, keratin filament, and supramolecular fiber, showed relatively lower representation in CRC compared to NCCs (28, 29), reflecting alterations in structural protein composition.

Within the MF category, activated terms were dominated by protease inhibitory and regulatory activities, including enzyme inhibitor activity, serine-type endopeptidase inhibitor activity, and peptidase regulatory activity (**Figure 4C**). These findings point to enhanced regulatory mechanisms controlling proteolytic processes in CRC saliva (31, 32). In contrast, functions associated with cytoskeletal binding and structural molecule activity, such as actin binding, cytoskeletal protein binding, and structural constituent of cytoskeleton (**Figure 4c**), were relatively decreased in CRC compared to NCCs (28, 29).

### A highly interconnected immune–metabolic signaling architecture characterizes CRC saliva

**Figure 5:**
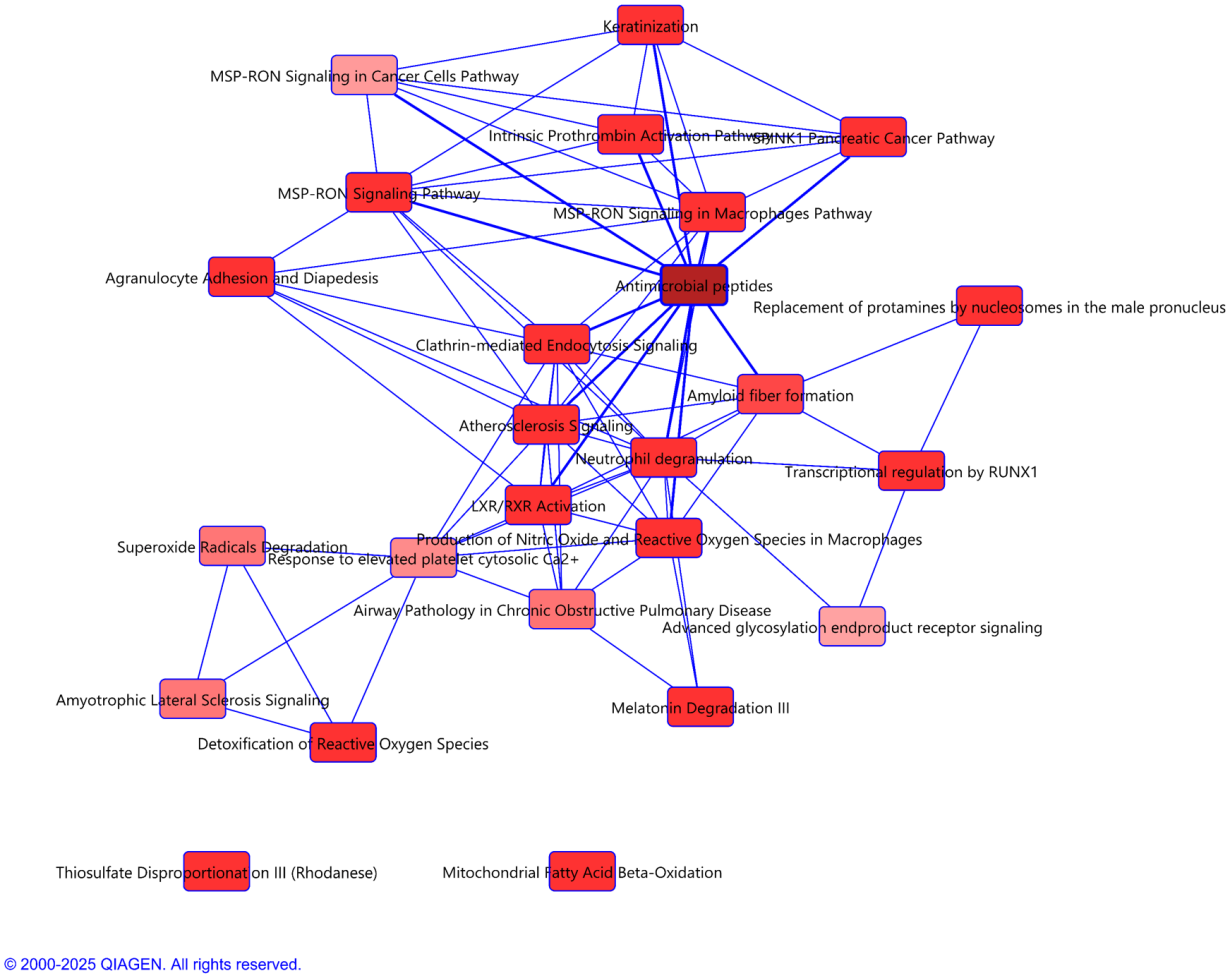
Overlapping Canonical Pathways Network generated using Ingenuity Pathway Analysis (IPA). Red nodes represent significantly enriched canonical pathways (p < 0.05) identified in the salivary proteome of CRC patients versus non-cancer controls (NCCs). Color intensity corresponds to the significance of pathway enrichment (−log10 p-value). Blue edges denote shared molecular components (e.g., genes or proteins) between pathways, representing functional overlap or regulatory crosstalk. The network highlights interrelated immune, metabolic, and signaling pathways implicated in CRC biology. Pathways such as MSP-RON signaling, neutrophil degranulation, antimicrobial peptides, and reactive oxygen species (ROS) detoxification formed core hubs within the network.

To further elucidate the functional interplay between dysregulated biological processes in CRC patients, we applied Ingenuity Pathway Analysis (IPA) “Overlapping Canonical Pathways” network analysis (**Figure 5**). This analysis revealed a highly interconnected signaling architecture among significantly enriched pathways in CRC saliva. Central nodes within the network included Antimicrobial Peptides, Clathrin-Mediated Endocytosis Signaling, Neutrophil Degranulation, and MSP-RON Signaling, all of which are implicated in inflammation, immune modulation, and cancer biology (33, 34). Metabolic pathways such as Reactive Oxygen Species (ROS) Detoxification, Fatty-acid β-Oxidation, and Melatonin Degradation III were also integrated into the network, suggesting a link between metabolic stress and tumorigenesis (35).

## Discussion

This study presents a comprehensive analysis of the salivary proteome in Iranian CRC patients compared with NCCs, revealing a distinct set of differentially abundant proteins with potential diagnostic relevance. By integrating demographic, lifestyle, and clinical characteristics with proteomic profiling and bioinformatics analyses, we identified coordinated alterations in immune-related, metabolic, and structural protein pathways. These findings not only reflect the systemic biological impact of CRC but also support the potential utility of saliva as a non-invasive source of disease-associated molecular signatures.

### Demographic and lifestyle factors

Between-group comparisons revealed significant differences in age, profession, family history, disease or surgical history, and physical activity levels (**Table 1**). CRC patients were significantly older than NCCs, consistent with the known age distribution of CRC incidence. Lower physical activity levels were more frequently reported in the CRC group, in line with previous observations describing reduced activity levels among CRC patients (36). A higher proportion of CRC patients reported a family history and prior disease or surgical history within this cohort. According to the predefined study criteria, individuals with known hereditary CRC syndromes were excluded; thus, “family history” refers to self-reported familial occurrence without documented hereditary cancer syndromes. No statistically significant differences were observed in dietary pattern, smoking status, alcohol consumption, viral infection status, regular probiotic use, or antibiotic usage. Although dietary factors have been widely discussed in relation to CRC risk (37), dietary patterns did not differ significantly between CRC patients and NCCs in this study. Given the exclusion of participants with recent extensive antibiotic or probiotic exposure (with the exception of one short-term antibiotic case and one short-term probiotic case), variation in these variables was minimal within the cohort. Overall, major lifestyle-related factors were broadly comparable between groups.

### Salivary proteome alterations and key biomarker candidates

Unsupervised hierarchical clustering of salivary proteomic profiles (**Figure 1**) demonstrated partial separation between CRC stage I patients and NCCs. Although complete segregation was not observed, several clusters showed visible alignment with clinical and demographic annotations, indicating inter-individual heterogeneity within both groups. This pattern suggests that salivary proteomic alterations in CRC coexist with background variability related to host characteristics.

Global differential abundance analysis using a covariate-adjusted volcano plot (**Figure 2**) identified a subset of proteins significantly altered between CRC and NCC after adjustment for age, sex, and smoking status. Several proteins exhibited increased abundance in CRC saliva, including members of the SPRR family (SPRR1A, SPRR3, and SPRR2D), as well as TXNDC17, DBI, PI3 (Elafin), KLK6, WFDC2, and FABP5. Among these, the consistent elevation of SPRR family members is notable. SPRR proteins have been linked to epithelial differentiation, epithelial-to-mesenchymal transition (EMT), and tumor progression in CRC and other epithelial malignancies (38–41). Their increased abundance in saliva may reflect tumor-associated epithelial remodeling and inflammatory signaling processes (42–44). Similarly, KLK6 has been associated with protease activity and metastatic behavior in CRC (45), while DBI and FABP5 are involved in lipid metabolism and metabolic regulation (46). PI3 (Elafin) and WFDC2 have been connected to inflammatory and extracellular matrix–related mechanisms in cancer biology (47, 48). Collectively, these findings indicate coordinated alterations in epithelial integrity, protease regulation, metabolism, and inflammatory signaling in CRC saliva. In addition to elevated proteins, several proteins showed reduced abundance in CRC saliva, including myeloperoxidase (MPO), lysozyme (LYZ), histone cluster 1 H2B family member D (HIST1H2BD), annexin A3 (ANXA3), and acyl-CoA dehydrogenase very long chain (ACADVL). MPO and LYZ are central components of innate immune defense, while histone-associated proteins are linked to chromatin organization and cellular turnover processes (49–53). Their decreased representation in CRC saliva may reflect alterations in immune-related pathways or epithelial homeostasis within the disease context. Subgroup analyses (**Figure 3**) further demonstrated variability in the prominence of selected proteins across demographic strata. Carcinoembryonic antigen-related cell adhesion molecule 5 (CEACAM5), cystatin B (CSTB), superoxide dismutase 1 (SOD1), DBI, and FABP5 showed stronger differential signals in males, whereas SPRR1A, SPRR3, small proline-rich protein 2A (SPRR2A), WFDC2, and TXNDC17 were more prominent in females. In middle-aged individuals, several SPRR family members and additional CRC-associated proteins remained differentially abundant between CRC patients and NCCs. These observations suggest that although demographic variables may influence specific salivary protein abundance patterns, multiple CRC-associated proteomic alterations remained detectable across demographic subgroups (54, 55). Overall, the identified salivary alterations support the presence of a multi-protein CRC-associated signature encompassing epithelial structural proteins, metabolic enzymes, immune-related mediators, and protease regulators. This integrated pattern reinforces the potential advantage of multiplex biomarker panels over single-marker approaches in non-invasive CRC detection (56, 57).

### Comparative analysis with other studies

To contextualize our findings, we compared the salivary biomarker candidates identified in this study with previously reported markers in CRC and other oro-GI malignancies, including OSCC, as summarized in **Tables 2** and **3**. Several proteins detected in our cohort have also been described in CRC tissue or serum studies, as well as in other GI cancers, supporting their broader involvement in oncogenic and inflammatory processes. At the same time, the specific combination and relative abundance patterns observed in our cohort suggest the presence of a CRC-associated salivary proteomic profile, rather than reliance on a single disease-specific marker (58). These findings highlight the importance of further validation in independent cohorts to determine the reproducibility and diagnostic specificity of this candidate panel. Recent molecular classification efforts in CRC, such as the genome- and transcriptome-based subtyping approach described by Guinney et al. (2015), further emphasize the biological heterogeneity of CRC and the value of integrating multi-layer molecular data (59). In this context, salivary proteomic signatures may represent a complementary, non-invasive dimension for refining CRC characterization.

### Functional enrichment: GSEA and IPA insights

Gene Set Enrichment Analysis (GSEA) (**Figure 4**) highlights a coordinated shift in structural and regulatory functions in the salivary proteome of CRC patients. The consistent suppression of cytoskeleton- and intermediate filament–related biological processes and cellular components across all three GO categories indicates a loss of structural organization, reflecting cytoskeletal remodeling commonly associated with malignant transformation (28–30). In parallel, the enrichment of protease inhibitory and regulatory molecular functions suggests altered control of proteolytic activity, potentially reflecting compensatory or tumor-associated mechanisms rather than generalized proteolysis (31, 32). Together, these patterns point to a saliva-based proteomic signature characterized by disrupted cellular architecture and enhanced protease regulation in CRC, underscoring the relevance of cytoskeletal integrity and proteolytic balance as hallmarks of disease-associated molecular alterations captured through non-invasive saliva sampling.

IPA-based canonical pathway analysis (**Figure 5**) revealed a network of interconnected immune and metabolic pathways in CRC saliva. Immune-related hubs included Neutrophil Degranulation, Antimicrobial Peptides, and MSP-RON Signaling, highlighting coordinated inflammatory and immune-modulatory activity (33, 34). Concurrent enrichment of Reactive Oxygen Species (ROS) Detoxification and Fatty-acid β-Oxidation pathways indicates altered oxidative balance and metabolic adaptation, processes commonly associated with CRC biology (33–35). The presence of Melatonin Degradation within the enriched network further supports the involvement of metabolic regulatory mechanisms (60). Overall, this interconnected map reflects a multifaceted CRC-associated signaling landscape in saliva, where immune-related pathways, oxidative stress mechanisms, and metabolic regulatory processes appear concurrently represented within a shared molecular network (35, 61).

### Limitations and future directions

Despite the robustness of our findings, several limitations should be acknowledged. The relatively modest sample size and lack of longitudinal data may limit the generalizability of the results. In addition, this study should be regarded as a discovery-phase pilot biomarker investigation intended to identify candidate salivary proteins associated with CRC. While encouraging differences were observed, future studies will require orthogonal validation and independent external cohorts to confirm reproducibility and clinical utility. Future investigations should include larger, multi-ethnic cohorts and consider integrating salivary proteomics with genomic, transcriptomic, and microbiome data to more comprehensively capture CRC complexity. Furthermore, the development of targeted proteomics platforms, such as selected reaction monitoring (SRM), may facilitate biomarker validation and support future clinical translation (62).

## Conclusions

This study underscores the diagnostic potential of salivary proteomics in CRC. The observed dual signature, characterized by suppression of cytoskeletal and epithelial structural programs alongside activation of immune and metabolic pathways, reflects the systemic nature of CRC and the informative value of saliva. With further validation, the identified biomarker candidates may contribute to the development of non-invasive diagnostic platforms and support more personalized approaches in CRC management.

## Data Availability

The mass spectrometry proteomics data have been deposited to the PRIDE repository via the ProteomeXchange Consortium under accession number PXD078610. Other datasets generated and/or analyzed during the current study are available from the corresponding author up-on reasonable request.

## References

[1] Siegel RL Wagle NS Cercek A Smith RA Jemal A . Colorectal cancer statistics, 2023. CA Cancer J Clin. (2023) 73:233–54. doi: 10.3322/caac.21772 36856579

[2] Xi Y Xu P . Global colorectal cancer burden in 2020 and projections to 2040. Transl Oncol. (2021) 14:101174. doi: 10.1016/j.tranon.2021.101174 34243011 PMC8273208

[3] Zeller G Tap J Voigt AY Sunagawa S Kultima JR Costea PI . Potential of fecal microbiota for early-stage detection of colorectal cancer. Mol Syst Biol. (2014) 10:766. doi: 10.15252/msb.20145645 25432777 PMC4299606

[4] Siskova A Cervena K Kral J Hucl T Vodicka P Vymetalkova V . Colorectal adenomas-genetics and searching for new molecular screening biomarkers. Int J Mol Sci. (2020) 21. doi: 10.3390/ijms21093260 32380676 PMC7247353

[5] Bosch S Acharjee A Quraishi MN Bijnsdorp IV Rojas P Bakkali A . Integration of stool microbiota, proteome and amino acid profiles to discriminate patients with adenomas and colorectal cancer. Gut Microbes. (2022) 14:2139979. doi: 10.1080/19490976.2022.2139979 36369736 PMC9662191

[6] Sivaram S Majumdar G Perin D Nessa A Broeders M Lynge E . Population-based cancer screening programmes in low-income and middle-income countries: regional consultation of the International Cancer Screening Network in India. Lancet Oncol. (2018) 19:e113–22. doi: 10.1016/s1470-2045(18)30003-2 29413465 PMC5835355

[7] Shaukat A Levin TR . Current and future colorectal cancer screening strategies. Nat Rev Gastroenterol Hepatol. (2022) 19:521–31. doi: 10.1038/s41575-022-00612-y 35505243 PMC9063618

[8] Kortlever TL van der Vlugt M Dekker E . Future of colorectal cancer screening: From one-size-FITs-all to tailor-made. In: Kortlever TL van der Vlugt M Dekker E , editors. Frontiers in Gastroenterology. (Lausanne, Switzerland: Lausanne, Switzerland) (2022). 10.3389/fgstr.2022.906052 PMC12952459 41822079

[9] Malathi N Mythili S Vasanthi HR . Salivary diagnostics: a brief review. ISRN Dent. (2014) 2014:158786. doi: 10.1155/2014/158786 24616813 PMC3926256

[10] Loo JA Yan W Ramachandran P Wong DT . Comparative human salivary and plasma proteomes. J Dent Res. (2010) 89:1016–23. doi: 10.1177/0022034510380414 20739693 PMC3144065

[11] Yoshizawa JM Schafer CA Schafer JJ Farrell JJ Paster BJ Wong DT . Salivary biomarkers: toward future clinical and diagnostic utilities. Clin Microbiol Rev. (2013) 26:781–91. doi: 10.1128/cmr.00021-13 24092855 PMC3811231

[12] Grassl N Kulak NA Pichler G Geyer PE Jung J Schubert S . Ultra-deep and quantitative saliva proteome reveals dynamics of the oral microbiome. Genome Med. (2016) 8:44. doi: 10.1186/s13073-016-0293-0 27102203 PMC4841045

[13] Farrell JJ Zhang L Zhou H Chia D Elashoff D Akin D . Variations of oral microbiota are associated with pancreatic diseases including pancreatic cancer. Gut. (2012) 61:582–8. doi: 10.1136/gutjnl-2011-300784 21994333 PMC3705763

[14] Granato DC Neves LX Trino LD Carnielli CM Lopes AFB Yokoo S . Meta-omics analysis indicates the saliva microbiome and its proteins associated with the prognosis of oral cancer patients. Biochim Biophys Acta Proteins Proteom. (2021) 1869:140659. doi: 10.1016/j.bbapap.2021.140659 33839314

[15] Zhao Y Wang C Goel A . Role of gut microbiota in epigenetic regulation of colorectal cancer. Biochim Biophys Acta Rev Cancer. (2021) 1875:188490. doi: 10.1016/j.bbcan.2020.188490 33321173 PMC7856101

[16] Sabit H Cevik E Tombuloglu H . Colorectal cancer: The epigenetic role of microbiome. World J Clin cases. (2019) 7:3683–97. doi: 10.12998/wjcc.v7.i22.3683 31799293 PMC6887622

[17] Rezasoltani S Azizmohammad Looha M Asadzadeh Aghdaei H Jasemi S Sechi LA Gazouli M . 16S rRNA sequencing analysis of the oral and fecal microbiota in colorectal cancer positives versus colorectal cancer negatives in Iranian population. Gut Pathog. (2024) 16:9. doi: 10.1186/s13099-024-00604-0 38378690 PMC10880352

[18] Rezasoltani S Aghdaei HA Jasemi S Gazouli M Dovrolis N Sadeghi A . Oral microbiota as novel biomarkers for colorectal cancer screening. Cancers. (2023) 15:192. doi: 10.3390/cancers15010192 36612188 PMC9818409

[19] Roca C Alkhateeb AA Deanhardt BK Macdonald JK Chi DL Wang JR . Saliva sampling method influences oral microbiome composition and taxa distribution associated with oral diseases. PloS One. (2024) 19:e0301016. doi: 10.1371/journal.pone.0301016 38547181 PMC10977688

[20] Hughes CS Moggridge S Müller T Sorensen PH Morin GB Krijgsveld J . Single-pot, solid-phase-enhanced sample preparation for proteomics experiments. Nat Protoc. (2019) 14:68–85. doi: 10.1038/s41596-018-0082-x 30464214

[21] Carnielli CM Macedo CCS De Rossi T Granato D Rivera C Domingues RR . Combining discovery and targeted proteomics reveals a prognostic signature in oral cancer. Nat Commun. (2018) 9. doi: 10.1038/s41467-018-05696-2 30185791 PMC6125363

[22] Villén J Gygi SP . The SCX/IMAC enrichment approach for global phosphorylation analysis by mass spectrometry. Nat Protoc. (2008) 3:1630–8. doi: 10.1038/nprot.2008.150 PMC2728452 18833199

[23] Voß H Moritz M Pelczar P Gagliani N Huber S Nippert V . Tissue sampling and homogenization with NIRL enables spatially resolved cell layer specific proteomic analysis of the murine intestine. Int J Mol Sci. (2022) 23. 10.3390/ijms23116132 PMC9181169 35682811

[24] Voß H Schlumbohm S Barwikowski P Wurlitzer M Dottermusch M Neumann P . HarmonizR enables data harmonization across independent proteomic datasets with appropriate handling of missing values. Nat Commun. (2022) 13:3523. doi: 10.1038/s41467-022-31007-x 35725563 PMC9209422

[25] Schumann Y Schlumbohm S Neumann JE Neumann P . High performance data integration for large-scale analyses of incomplete omic profiles using batch-effect reduction trees (BERT). Nat Commun. (2025) 16:7104. doi: 10.1038/s41467-025-62237-4 40753179 PMC12318123

[26] Yu G Wang LG Han Y He QY . clusterProfiler: an R package for comparing biological themes among gene clusters. Omics. (2012) 16:284–7. doi: 10.1089/omi.2011.0118 22455463 PMC3339379

[27] Yang D Li R Xia J Li W Ma L Ye L . Long noncoding RNA PCAT18 upregulates SPRR3 to promote colorectal cancer progression by binding to miR-759. Cancer Manage Res. (2020) 12:11445–52. doi: 10.2147/cmar.s272652 33204157 PMC7667148

[28] Fife CM McCarroll JA Kavallaris M . Movers and shakers: cell cytoskeleton in cancer metastasis. Br J Pharmacol. (2014) 171:5507–23. doi: 10.1111/bph.12704 24665826 PMC4290699

[29] Fletcher DA Mullins RD . Cell mechanics and the cytoskeleton. Nature. (2010) 463:485–92. doi: 10.1038/nature08908 20110992 PMC2851742

[30] Windoffer R Beil M Magin TM Leube RE . Cytoskeleton in motion: the dynamics of keratin intermediate filaments in epithelia. J Cell Biol. (2011) 194:669–78. doi: 10.1083/jcb.201008095 21893596 PMC3171125

[31] Mason SD Joyce JA . Proteolytic networks in cancer. Trends Cell Biol. (2011) 21:228–37. doi: 10.1016/j.tcb.2010.12.002 21232958 PMC3840715

[32] Eatemadi A Aiyelabegan HT Negahdari B Mazlomi MA Daraee H Daraee N . Role of protease and protease inhibitors in cancer pathogenesis and treatment. BioMed Pharmacother. (2017) 86:221–31. doi: 10.1016/j.biopha.2016.12.021 28006747

[33] Huang L Fang X Shi D Yao S Wu W Fang Q . MSP-RON pathway: potential regulator of inflammation and innate immunity. Front Immunol. (2020) 11:569082. doi: 10.3389/fimmu.2020.569082 33117355 PMC7577085

[34] Wang X He S Gong X Lei S Zhang Q Xiong J . Neutrophils in colorectal cancer: mechanisms, prognostic value, and therapeutic implications. Front Immunol. (2025) 16:2025. doi: 10.3389/fimmu.2025.1538635 40092983 PMC11906667

[35] Catalano T Selvaggi F Cotellese R Aceto GM . The role of reactive oxygen species in colorectal cancer initiation and progression: perspectives on theranostic approaches. Cancers. (2025) 17:752. doi: 10.3390/cancers17050752 40075600 PMC11899472

[36] Wang Q Zhou W . Roles and molecular mechanisms of physical exercise in cancer prevention and treatment. J Sport Health Sci. (2021) 10:201–10. doi: 10.1016/j.jshs.2020.07.008 32738520 PMC7987556

[37] Mehta RS Song M Nishihara R Drew DA Wu K Qian ZR . Dietary patterns and risk of colorectal cancer: analysis by tumor location and molecular subtypes. Gastroenterology. (2017) 152:1944–53.e1. doi: 10.1016/s0016-5085(17)30805-3 28249812 PMC5447483

[38] Chaudhary RK Patil P Shetty VV L A Shetty Kalladka S Mateti UV . Decoding the role of SPRR1A and SPRR1B gene in cancer: a comprehensive review. Gene Rep. (2024) 36:101926. doi: 10.1016/j.genrep.2024.101926 38826717

[39] Cho D-H Jo YK Roh SA Na Y-S Kim TW Jang SJ . Upregulation of SPRR3 promotes colorectal tumorigenesis. Mol Med. (2010) 16:271–7. doi: 10.2119/molmed.2009.00187 20379613 PMC2896463

[40] Xu X Wei S Chen Y Yu D Wang X Dong X . Serum small proline-rich protein 2A (SPRR2A) is a noninvasive biomarker in gastric cancer. Dis Markers. (2020) 2020:8493796. doi: 10.1155/2020/8493796 32908616 PMC7475742

[41] Carregaro F Stefanini AC Henrique T Tajara EH . Study of small proline-rich proteins (SPRRs) in health and disease: a review of the literature. Arch Dermatol Res. (2013) 305:857–66. doi: 10.1007/s00403-013-1415-9 24085571

[42] Kong X Wang D Sun W Chen M Chen J Shi J . Small proline-rich protein 2A and 2D are regulated by the RBM38-p73 axis and associated with p73-dependent suppression of chronic inflammation. Cancers (Basel). (2021) 13. doi: 10.3390/cancers13112829 34204113 PMC8201237

[43] Kotelevets L Chastre E . Extracellular vesicles in colorectal cancer: from tumor growth and metastasis to biomarkers and nanomedications. Cancers. (2023) 15. doi: 10.3390/cancers15041107 36831450 PMC9953945

[44] Wu J Liu G Jia R Guo J . Salivary extracellular vesicles: biomarkers and beyond in human diseases. Int J Mol Sci. (2023) 24. doi: 10.3390/ijms242417328 38139157 PMC10743646

[45] Bouzid H Soualmia F Oikonomopoulou K Soosaipillai A Walker F Louati K . Kallikrein-related peptidase 6 (KLK6) as a contributor toward an aggressive cancer cell phenotype: a potential role in colon cancer peritoneal metastasis. Biomolecules. (2022) 12. doi: 10.3390/biom12071003 35883559 PMC9312869

[46] Gokce M Velioglu HA Bektay MY Guler EM . Evaluating the clinical significance of diazepam binding inhibitor in Alzheimer's disease: a comparison with inflammatory, oxidative, and neurodegenerative biomarkers. Gerontology. (2023) 69:1104–12. doi: 10.1159/000531849 37607528

[47] Wang C Liao Y He W Zhang H Zuo D Liu W . Elafin promotes tumour metastasis and attenuates the anti-metastatic effects of erlotinib via binding to EGFR in hepatocellular carcinoma. J Exp Clin Cancer Res. (2021) 40:113. doi: 10.1186/s13046-021-01904-y 33771199 PMC7995733

[48] James NE Gura M Woodman M Freiman RN Ribeiro JR . A bioinformatic analysis of WFDC2 (HE4) expression in high grade serous ovarian cancer reveals tumor-specific changes in metabolic and extracellular matrix gene expression. Med Oncol. (2022) 39:71. doi: 10.1007/s12032-022-01665-4 35568777 PMC9107348

[49] Martín-García D García-Aranda M Redondo M . Biomarker identification through proteomics in colorectal cancer. Int J Mol Sci. (2024) 25. doi: 10.3390/ijms25042283 PMC10888664 38396959

[50] Rydzynska Z Pawlik B Krzyzanowski D Mlynarski W Madzio J . Neutrophil elastase defects in congenital neutropenia. Front Immunol. (2021) 12:653932. doi: 10.3389/fimmu.2021.653932 33968054 PMC8100030

[51] Ferraboschi P Ciceri S Grisenti P . Applications of lysozyme, an innate immune defense factor, as an alternative antibiotic. Antibiotics (Basel). (2021) 10. doi: 10.3390/antibiotics10121534 34943746 PMC8698798

[52] Kawasaki H Iwamuro S . Potential roles of histones in host defense as antimicrobial agents. Infect Disord Drug Targets. (2008) 8:195–205. doi: 10.2174/1871526510808030195 18782037

[53] Wang H Mao X Ye L Cheng H Dai X . The role of the S100 protein family in glioma. J Cancer. (2022) 13:3022–30. doi: 10.7150/jca.73365 36046652 PMC9414020

[54] Gong Y Liu Y Wang T Li Z Gao L Chen H . Age-associated proteomic signatures and potential clinically actionable targets of colorectal cancer. Mol Cell Proteomics. (2021) 20:100115. doi: 10.1016/j.mcpro.2021.100115 34129943 PMC8441843

[55] Su H Gu X Zhang W Lin F Lu X Zeng X . Identification of salivary biomarkers in colorectal cancer by integrating Olink proteomics and metabolomics. J Proteome Res. (2025) 24:2542–52. doi: 10.1021/acs.jproteome.5c00091 40183281 PMC12054530

[56] Pellino G Gallo G Pallante P Capasso R De Stefano A Maretto I . Noninvasive biomarkers of colorectal cancer: Role in diagnosis and personalised treatment perspectives. Gastroenterol Res Pract. (2018) 2018:2397863. doi: 10.1155/2018/2397863 30008744 PMC6020538

[57] Rezasoltani S Sharafkhah M Asadzadeh Aghdaei H Nazemalhosseini Mojarad E Dabiri H Akhavan Sepahi A . Applying simple linear combination, multiple logistic and factor analysis methods for candidate fecal bacteria as novel biomarkers for early detection of adenomatous polyps and colon cancer. J Microbiol Methods. (2018) 155:82–8. doi: 10.1016/j.mimet.2018.11.007 30439465

[58] Duffy MJ van Dalen A Haglund C Hansson L Holinski-Feder E Klapdor R . Tumour markers in colorectal cancer: European Group on Tumour Markers (EGTM) guidelines for clinical use. Eur J Cancer. (2007) 43:1348–60. doi: 10.1016/j.ejca.2007.03.021 17512720

[59] Guinney J Dienstmann R Wang X de Reyniès A Schlicker A Soneson C . The consensus molecular subtypes of colorectal cancer. Nat Med. (2015) 21:1350–6. doi: 10.1038/nrc.2016.126 26457759 PMC4636487

[60] Reiter RJ Rosales-Corral S Tan DX Jou MJ Galano A Xu B . Melatonin as a mitochondria-targeted antioxidant: one of evolution's best ideas. Cell Mol Life Sci. (2017) 74:3863–81. doi: 10.1007/s00018-017-2609-7 28864909 PMC11107735

[61] Yuan K Hu D Mo X Zeng R Wu B Zhang Z . Novel diagnostic biomarkers of oxidative stress, immune- infiltration characteristics and experimental validation of SERPINE1 in colon cancer. Discover Oncol. (2023) 14:206. doi: 10.1007/s12672-023-00833-w 37980291 PMC10657345

[62] Birhanu AG . Mass spectrometry-based proteomics as an emerging tool in clinical laboratories. Clin Proteomics. (2023) 20:32. doi: 10.1186/s12014-023-09424-x 37633929 PMC10464495

[63] Deng Y Zheng X Zhang Y Xu M Ye C Lin M . High SPRR1A expression is associated with poor survival in patients with colon cancer. Oncol Lett. (2020) 19:3417–24. doi: 10.3892/ol.2020.11453 32269614 PMC7115157

[64] Abdelmaksoud NM Abulsoud AI Abdelghany TM Elshaer SS Rizk SM Senousy MA . Mitochondrial remodeling in colorectal cancer initiation, progression, metastasis, and therapy: A review. Pathol Res Pract. (2023) 246:154509. doi: 10.1016/j.prp.2023.154509 37182313

[65] Tieng FYF Abu N Sukor S Mohd Azman ZA Mahamad Nadzir N Lee LH . L1CAM, CA9, KLK6, HPN, and ALDH1A1 as potential serum markers in primary and metastatic colorectal cancer screening. Diagnostics (Basel). (2020) 10. doi: 10.3390/diagnostics10070444 32630086 PMC7400057

[66] Chen L Shu P Zhang X Ye S Tian L Shen S . S100A8-mediated inflammatory signaling drives colorectal cancer progression via the CXCL5/CXCR2 axis. J Cancer. (2024) 15:3452–65. doi: 10.7150/jca.92588 38817853 PMC11134430

[67] Bayo Calero J Castaño López MA Casado Monge PG Díaz Portillo J Bejarano García A Navarro Roldán F . Analysis of blood markers for early colorectal cancer diagnosis. J Gastrointestinal Oncol. (2022) 13:2259–68. doi: 10.21037/jgo-21-747 36388660 PMC9660082

[68] Warsinggih Irawan B Labeda I Lusikooy RE Sampetoding S Kusuma MI . Association of superoxide dismutase enzyme with staging and grade of differentiation colorectal cancer: A cross-sectional study. Ann Med Surg (Lond). (2020) 58:194–9. doi: 10.1016/j.amsu.2020.08.032 32994983 PMC7505864

[69] Acevedo-León D Monzó-Beltrán L Pérez-Sánchez L Naranjo-Morillo E Gómez-Abril S Estañ-Capell N . Oxidative stress and DNA damage markers in colorectal cancer. Int J Mol Sci. (2022) 23. doi: 10.3390/ijms231911664 36232966 PMC9569897

[70] Liu Y Tian Y Wu T Dai Y Wang W Teng G . High expression and clinical significance of elafin in colorectal cancer. Gastroenterol Res Pract. (2019) 2019:4946824. doi: 10.1155/2019/4946824 31281349 PMC6590570

[71] Benelli R Costa D Mastracci L Grillo F Olsen MJ Barboro P . Aspartate-β-hydroxylase: a promising target to limit the local invasiveness of colorectal cancer. Cancers (Basel). (2020) 12. doi: 10.3390/cancers12040971 32295249 PMC7226058

[72] Ahuja US Puri N Bagewadi A Keluskar V Ahuja A Singh HP . Comparative evaluation of serum alpha-1antitrypsin levels in patients with oral squamous cell carcinoma and in subjects with tobacco habit without carcinoma. J Family Med Prim Care. (2019) 8:3657–63. doi: 10.4103/jfmpc.jfmpc_571_19 31803669 PMC6881917

[73] Shiiba M Saito K Yamagami H Nakashima D Higo M Kasamatsu A . Interleukin-1 receptor antagonist (IL1RN) is associated with suppression of early carcinogenic events in human oral Malignancies. Int J Oncol. (2015) 46:1978–84. doi: 10.3892/ijo.2015.2917 25738940

[74] Ding Y Yi J Wang J Sun Z . Interleukin-1 receptor antagonist: a promising cytokine against human squamous cell carcinomas. Heliyon. (2023) 9:e14960. doi: 10.1016/j.heliyon.2023.e14960 37025835 PMC10070157

[75] Zhang J Shi Z Huang J Zou X . CSTB downregulation promotes cell proliferation and migration and suppresses apoptosis in gastric cancer SGC-7901 cell line. Oncol Res. (2016) 24:487–94. doi: 10.3727/096504016x14685034103752 28281969 PMC7838608

[76] Ouyang P Lin B Du J Pan H Yu H He R . Global gene expression analysis of knockdown triosephosphate isomerase (TPI) gene in human gastric cancer cell line MGC-803. Gene. (2018) 647:61–72. doi: 10.1016/j.gene.2018.01.014 29307852

[77] Cui Y Tian M Zong M Teng M Chen Y Lu J . Proteomic analysis of pancreatic ductal adenocarcinoma compared with normal adjacent pancreatic tissue and pancreatic benign cystadenoma. Pancreatology. (2009) 9:89–98. doi: 10.1159/000178879 19077459

[78] Guo C Liu S Greenaway F Sun MZ . Potential role of annexin A7 in cancers. Clin Chim Acta. (2013) 423:83–9. doi: 10.1016/j.cca.2013.04.018 23639634

[79] Feng H Feng J Han X Ying Y Lou W Liu L . The potential of Siglecs and sialic acids as biomarkers and therapeutic targets in tumor immunotherapy. Cancers. (2024) 16:289. doi: 10.3390/cancers16020289 38254780 PMC10813689

[80] Dinh NTM Nguyen TM Park MK Lee CH . Y-box binding protein 1: Unraveling the multifaceted role in cancer development and therapeutic potential. Int J Mol Sci. (2024) 25:717. doi: 10.3390/ijms25020717 38255791 PMC10815159

